# Single-cell RNA sequencing reveals common interactions between follicle immune cells and granulosa cells in premature ovarian insufficiency patients^†^

**DOI:** 10.1093/biolre/ioae157

**Published:** 2024-11-08

**Authors:** Ying Han, Junrong Diao, Xinyan Wang, Shuai Zhang, Lina Yuan, Yaqiong Ping, Ge Gao, Yunshan Zhang, Haining Luo

**Affiliations:** Tianjin Key Laboratory of Human Development and Reproductive Regulation, Tianjin Central Hospital of Obstetrics and Gynecology/Nankai University Affiliated Maternity Hospital, Tianjin, China; Tianjin Key Laboratory of Human Development and Reproductive Regulation, Tianjin Central Hospital of Obstetrics and Gynecology/Nankai University Affiliated Maternity Hospital, Tianjin, China; Tianjin Key Laboratory of Human Development and Reproductive Regulation, Tianjin Central Hospital of Obstetrics and Gynecology/Nankai University Affiliated Maternity Hospital, Tianjin, China; Tianjin Key Laboratory of Human Development and Reproductive Regulation, Tianjin Central Hospital of Obstetrics and Gynecology/Nankai University Affiliated Maternity Hospital, Tianjin, China; Tianjin Key Laboratory of Human Development and Reproductive Regulation, Tianjin Central Hospital of Obstetrics and Gynecology/Nankai University Affiliated Maternity Hospital, Tianjin, China; Tianjin Key Laboratory of Human Development and Reproductive Regulation, Tianjin Central Hospital of Obstetrics and Gynecology/Nankai University Affiliated Maternity Hospital, Tianjin, China; Tianjin Key Laboratory of Human Development and Reproductive Regulation, Tianjin Central Hospital of Obstetrics and Gynecology/Nankai University Affiliated Maternity Hospital, Tianjin, China; Tianjin Key Laboratory of Human Development and Reproductive Regulation, Tianjin Central Hospital of Obstetrics and Gynecology/Nankai University Affiliated Maternity Hospital, Tianjin, China; Tianjin Key Laboratory of Human Development and Reproductive Regulation, Tianjin Central Hospital of Obstetrics and Gynecology/Nankai University Affiliated Maternity Hospital, Tianjin, China

**Keywords:** single-cell RNA sequencing, follicle microenvironment, premature ovarian insufficiency, inflammation, VEGFA–FLT1

## Abstract

This study aims to investigate the follicle microenvironment of individuals with premature ovarian insufficiency (POI), normal ovarian reserve (normal), and advanced maternal age (AMA), and identify potential therapeutic targets. A total of nine women, including three POI, three normal, and three AMA women, who underwent in vitro fertilization or intracytoplasmic sperm injection were included in this study. For each participant, the first punctured follicle not containing cumulus cells were submitted to single-cell RNA sequencing to explore the characteristics of the follicle microenvironment of POI, normal, and AMA individuals. A total of 87,323 cells were isolated and grouped into six clusters: T cells, B cells, neutrophils, basophils, mononuclear phagocytes, and granulosa cells. Further analysis demonstrated that the population of granulosa cells in cluster 6 was increased in AMA and POI patients, whereas the population of gamma delta T (GDT) cells was decreased. We also found that the genes that were differentially expressed between GDT cells and monocytes were enriched in ribosome- and endoplasmic reticulum (ER)-related pathways. In addition, it showed that VEGFA–FLT1 interaction between the monocytes and granulosa cells may be lost in the AMA and POI patients as compared with the normal group. Loss of the VEGFA–FLT1 interaction in monocytes and granulosa cells, along with enriched ER- and ribosome-related pathways, may drive excess inflammation, accelerating granulosa cell senility and contributing to infertility. This study provides new insights into the pathogenesis of POI and aging and highlights the VEGFA–FLT1 interaction may be a potential therapeutic target for reducing inflammation and treating POI.

## Teaser

**Summary Sentence**

Composition of immune cells and granulosa cells of follicular fluid in POI patients, AMA, and normal individuals.

## Graphical Abstract

**Graphical Abstract:**
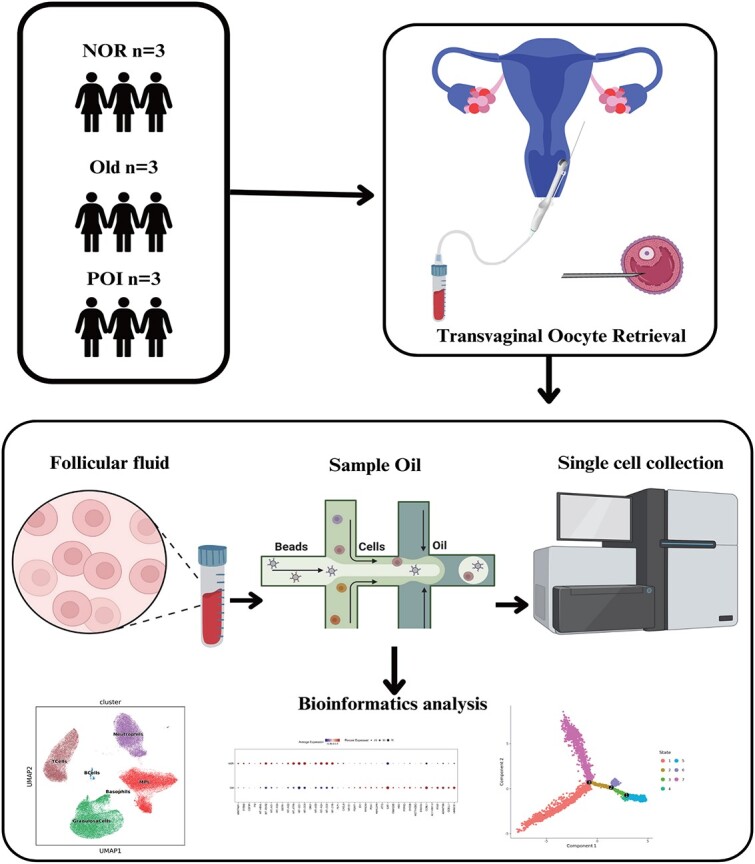

## Introduction

Premature ovarian insufficiency (POI) is defined of the depletion or dysfunction of ovarian follicles caused by reduced gonadal function in women under the age of 40 [1]. Premature ovarian insufficiency is a major cause of female infertility, and women with POI respond poorly to various ovulation induction protocols [2]. Additionally, POI increases the risk of osteoporosis and coronary heart disease, and its mortality rate is approximately twice that of the general population [3]. However, the etiology of POI is highly heterogeneous, and its pathogenesis remains unclear [4, 5].

The follicle microenvironment, which consists of cells and extracellular matrix components, is important for oocyte development and ovulation [6, 7]. For example, granulosa cells (GCs), which secrete cytokines, regulate meiosis in mammalian follicles before ovulation [8, 9]. The follicle microenvironment also contains a small number of immune cells, including macrophages such as the inflammatory (M1) macrophages and the anti-inflammatory (M2) macrophages, and lymphocytes, and granulocytes. These immune cells accumulate near the ovarian theca vasculature around the time of ovulation and then migrate to the developing corpus luteum. Ovarian immune cells are involved in modulating the process of oocyte development through phagocytosis and antigen presentation, the secretion of proteolytic enzymes for tissue remodeling, and the production of cytokines, chemokines, and growth factors [10–12]. Recently, Wu et al. [13] used single-cell RNA sequencing (scRNA-seq) to investigate the follicular microenvironment of MII oocytes in six human preovulatory follicles. They demonstrated that a specific subcluster of terminal GCs, with high levels of adhesion molecules, promotes macrophage recruitment and residence, thereby contributing to the heterogeneity of the immune cell composition in preovulatory follicles.

Here, we performed scRNA-seq on follicular fluid from normal individuals, AMA individuals, and POI patients to examine differences in the follicle microenvironment and identify potential therapeutic targets for POI.

## Materials and methods

### Study population

Three follicular fluid samples were collected from three POI patients who underwent in vitro fertilization (IVF)/intracytoplasmic sperm injection (ICSI) at the Tianjin Central Hospital of Obstetrics and Gynecology/Nankai University Affiliated Maternity Hospital (Tianjin, China). All patients underwent controlled ovarian stimulation using an antagonist protocol. Patients with ovarian dysfunction caused by factors such as ovarian surgery or medication were excluded from our study. Additionally, six discarded follicular fluid samples were obtained from three normal and three AMA individuals. The normal group included the individuals with reproductive age and normal ovarian reserve function, and underwent IVF or ICSI due to the fallopian tube damage or male factor. The AMA group included the individuals with advanced maternal age (≥40 years old). The diagnostic criteria for POI were (1) age < 40 years, (2) oligomenorrhea or amenorrhea for at least 4 months, and (3) at least two test results showing a basal follicle-stimulating hormone (bFSH) level of >25 IU/L (testing interval > 4 weeks). Individuals with type I or type II diabetes; impaired thyroid, renal, or liver function; congenital adrenal hyperplasia; endometriosis; hypothalamic amenorrhea; or chromosomal abnormalities were excluded from the study. Ethical approval for the study was granted by the institutional review board of the Reproductive Center of Tianjin Central Hospital of Obstetrics and Gynecology/Nankai University Affiliated Maternity Hospital (approval no. 2022KY020).

### Sample collection and scRNA-seq library preparation

For each participant, the first punctured follicle (diameter: 18–20 mm) including cumulus cells, the main components of the cumulus–oocyte complexes, was collected. Then, the cumulus cells were removed through gentle pipetting in 1% hyaluronidase, followed by washing with phosphate-buffered saline (PBS). The cells were then resuspended in medium for preparation and handing of gametes and for in vitro fertilisation (G-IVF) (Vitrolife, Sweden). The follicular fluid cells besides cumulus cells isolated from the same follicle were subjected to scRNA-seq. The single-cell preparations were suspended in PBS at 1 × 10^5^ cells/mL and then transferred to a microfluidic chip (GEXSCOPE Single Cell RNA-seq Kit; Singleron Biotechnologies, Jiangsu, China). The scRNA-seq libraries were constructed (Singleron Biotechnologies) and sequenced on an Illumina HiSeq X10 instrument using 150 bp paired-end reads.

### Generation of single-cell gene expression matrices

The raw reads were processed to generate gene expression matrices using CeleScope (https://github.com/singleron-RD/CeleScope). First, low-quality reads, poly-A tails, and adaptor sequences were removed from the raw data. The reads were then aligned to the GRCh38 human reference genome (Ensembl version 92) using STAR (version 020201). Using featureCounts (version 1.6.2), reads from the same gene and cell barcodes were pooled together, and the number of unique molecular identifiers (UMIs) per gene per cell was counted. Cell selection was performed using the UMI-tools algorithm. The numbers and quality of the cells were evaluated to identify effective cells and evaluate their gene expression.

### Dimensionality reduction, unsupervised clustering, differential gene expression screening, and functional analyses

Cell normalization and filtering were performed using the Seurat package. Principal component analysis and t-distributed stochastic neighbor embedding were used to reduce data dimensionality and to describe relationships between single cells. For unsupervised clustering, K-nearest neighbors were calculated, and a shared nearest neighbor graph was constructed using FindNeighbor. FindClusters was then used to identify cell clusters through modularity optimization techniques using the Louvain algorithm. For example, the genes of *CD3D*, *KLRD1*, *NKG7*, *FCGR3A*, and *KLRF1* were used to identify NK cells [14–16]. The FindAllMarkers function of the Seurat package and the Wilcoxon rank-sum test (with *P* values adjusted for multiple testing using Bonferroni correction) were used to identify the differentially expressed genes (DEGs) in each cell type compared with other cell types. Significant DEGs were identified using a |fold change| of >0.25 and a false discovery rate of <0.05 as cutoff thresholds. The package clusterProfiler (3.14.3) was used to perform Gene Ontology (GO) analysis on the DEGs and significant biological processes were identified using the thresholds “pvalueCutoff = 0.05” and “qvalueCutoff = 0.05.”

### Single-cell RNA sequencing signature score

For gene scoring analysis, we compared different gene signatures in subpopulations using Seurat’s AddModuleScore function [17]. The immunosuppressive signature score was defined as the average expression of a series of immune checkpoint inhibitors [18] and immunosuppressive molecules [19], including *CD244*, *CD160*, *cytotoxic T-lymphocyte associated protein 4 (CTLA4)*, *programmed cell death 1 (PDCD1)*, *T cell immunoreceptor with Ig and ITIM domains (TIGIT)*, *layilin (LAYN)*, *lymphocyte activating 3 (LAG3)*, *hepatitis A virus cellular receptor 2 (HAVCR2)*, *CD274*, *CD47*, *CD96*, *ectonucleoside triphosphate diphosphohydrolase 1 (ENTPD1*), *V-set immunoregulatory receptor (VSIR)*, *B and T lymphocyte associated (BTLA)*, *Epstein-Barr virus induced 3 (EBI3)*, *interleukin 2 receptor subunit beta (IL2RB)*, *interleukin 2 receptor subunit alpha (IL2RA*), and *interleukin 2 receptor subunit gamma (IL2RG)*. The dysfunctional signature score [20] was defined by the average expression of *LAG3*, *HAVCR2*, *PDCD1*, *parathymosin (PTMS)*, *FAM3 metabolism regulating signaling molecule C (FAM3C)*, *interferon gamma (IFNG)*, *A-kinase anchoring protein 5 (AKAP5)*, *CD7*, *pleckstrin homology like domain family A member 1 (PHLDA1)*, *ectonucleoside triphosphate diphosphohydrolase 1 (ENTPD1)*, *synaptosome associated protein 47 (SNAP47)*, *tensin 3 (TNS3)*, *C-X-C motif chemokine ligand 13 (CXCL13)*, *retinol dehydrogenase 10 (RDH10)*, *diacylglycerol kinase eta (DGKH)*, *killer cell immunoglobulin like receptor, two Ig domains and long cytoplasmic tail 4 (KIR2DL4)*, *lysosomal trafficking regulator (LYST)*, *MIR155 host gene (MIR155HG)*, *RAB27A, member RAS oncogene family (RAB27A)*, *colony stimulating factor 1 (CSF1)*, *TNF receptor superfamily member 9 (TNFRSF9)*, *CTLA4*, *CD27*, *C-C motif chemokine ligand 3 (CCL3)*, *integrin subunit alpha E (ITGAE)*, *phosphoprotein membrane anchor with glycosphingolipid microdomains 1 (PAG1)*, *TNF receptor superfamily member 1B (TNFRSF1B)*, *polypeptide N-acetylgalactosaminyltransferase 1 (GALNT1)*, *guanylate binding protein 2 (GBP2)*, *myosin VIIA (MYO7A)*, and *T cell immunoreceptor with Ig and ITIM domains (TIGIT)*. A list of transcription factors [21] involved in T-cell exhaustion, including *basic leucine zipper ATF-like transcription factor (BATF)*, *BCL6 transcription repressor (BCL6)*, *basic helix-loop-helix family member e40 (BHLHE40)*, *BTLA*, *CD200*, *eomesodermin (EOMES)*, *ETS variant transcription factor 1 (ETV1)*, *forkhead box P3 (FOXP3)*, *hypoxia inducible factor 4 subunit alpha (HIF4A)*, *HOP homeobox (HOPX)*, *inhibitor of DNA binding 2 (ID2)*, *inhibitor of DNA binding 3 (ID3)*, *interferon gamma inducible protein 16 (IFI16)*, *IKAROS family zinc finger (IKZF)*, *IKAROS family zinc finger 1 (IKZF3)*, *nuclear receptor subfamily 4 group A member 1 (NR4A1)*, *nuclear receptor subfamily 4 group A member 2 (NR4A2)*, *nuclear receptor subfamily 4 group A member 3 (NR4A3)*, *PR/SET domain 1 (PRDM1)*, *recombination signal binding protein for immunoglobulin kappa J region (RBPJ)*, *SRY-box transcription factor 4 (SOX4)*, *signal transducer and activator of transcription 3 (STAT3)*, *T-box transcription factor 21 (TBX21)*, *transcription factor 7 (TCF7)*, *thymocyte selection associated high mobility group box (TOX)*, *TOX2*, *vitamin D receptor (VDR)*, *zinc finger BED-type containing 2 (ZBED2)*, *zinc finger protein 683 (ZNF683)*, *interferon gamma inducible protein 16 (IFI16)*, *DR1 associated protein 1 (DRAP1)*, and *ETS proto-oncogene 1, transcription factor (ETS1)*, was also included.

### Cell–cell interactions

Crosstalk analysis between various cell types was performed using the CellChat package (v.0.5.5) with CellChatDB.huma as the ligand–receptor interaction reference database. The function computeCommunProbPathway was used to infer cell–cell communication at the signaling pathway level. Interactions between signaling pathways in various cell subtypes were investigated using the identifyCommunicationPatterns function.

### Determination of the developmental trajectory

To determine potential lineage differentiation between different GC subtypes, we performed trajectory analysis using the Monocle 2 (version 2.18.0) [22] algorithm. To this end, a CellDataSet object was constructed using the newCellDataSet function with expressionFamily set as the negbinomial.size. Dimensionality reduction was performed using the DDRTree algorithm (max_components parameters = 4) based on the expression of the top 3000 highly variable genes. Next, the cell trajectory was described using the orderCells function. The inferred cell trajectories were then visualized using the plot_cell_trajectory function. To visualize genes whose expression levels changed along a pseudotime trajectory, we used the plot_pseudotime_heatmap function and transcription factor genes with an adjusted *P* value of <0.01 and a fold change of >1.5.

### Immunofluorescence

The granulosa cells were derived from the nine individuals as same as the scRNA-seq, three for each of POI, AMA, and normal groups. Then, the granulosa cells were fixed in 4% paraformaldehyde for 24 h, blocked with 2% bovine serum albumin for 1 h, and stained with an anti-FLT1 antibody (cat no. ab2350, 1:100 dilution; Abcam, USA) overnight at 4°C. The secondary antibody used was an Alexa Fluor 488-conjugated anti-rabbit donkey IgG (ab150073) (1:500; Abcam). Nuclei were labeled with DAPI (C1002; Beyotime, China). Images were acquired with an inverted fluorescence microscope (Olympus IX71, Tokyo, Japan) or Zeiss LSM 510 laser scanning microscope (Zeiss, Jena, Germany).

## Results

### Cellular constitution of human follicular fluid samples

**Figure 1:**
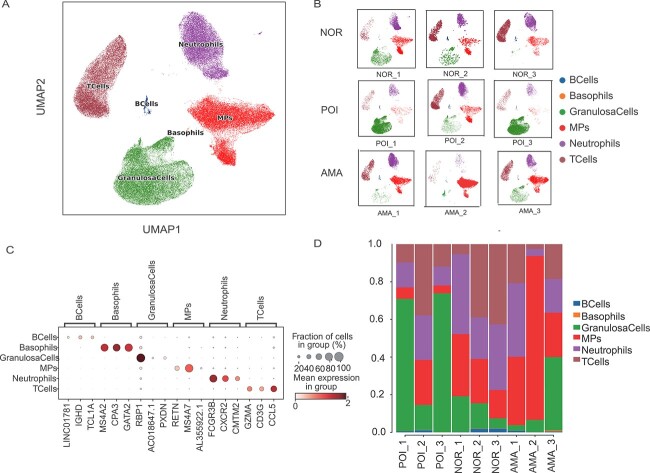
Single-cell atlas of follicular fluid samples from normal individuals, AMA individuals, and POI patients. (**A**) UMAP plot of the 87,323 cells from the nine samples based on the five cell clusters and (**B**) nine individuals. (**C**) Bubble plot of the canonical cell type markers of the five main cell types. (**D**) The distribution of the five main cell clusters in samples from normal individuals, AMA individuals, and POI patients.

To determine the pathogenesis of POI and its correlation with natural aging, we performed scRNA-seq on nine follicular fluid samples, three each from POI patients, AMA individuals, and normal individuals. The age range was between 28 and 32, 41 and 45, and 31 and 35 for the normal, AMA, and POI individuals. One individual of the normal group, two individuals of the AMA group, and one individual of the POI group received ICSI, respectively. Other clinical data including body mass index, bFSH, anti-Müllerian hormone, and number of oocytes are shown in Table 1. A total of 87,323 cells were isolated and divided into T cell, B cell, neutrophil, basophil, mononuclear phagocyte (MP), and GC clusters (Figure 1A). UMAP was used to reveal the distribution of these six clusters in each of the nine individuals (Figure 1B). This analysis revealed that B cells expressed high levels of *IGHD* and *TCL1A*; basophils expressed high levels of *MS4A2*, *GPA3*, and *GATA2*; granulosa cells expressed high levels of *RBP1*; MPs expressed *MS4A7* and *RETN*; neutrophils expressed *FCGR3B*, *CXCR2*, and *GMTM2*; and T cells expressed high levels of *CCL5*, *GZMA*, and *CD3G* (Figure 1C). Comparison of the proportions of these six clusters in samples from POI patients, AMA individuals, and normal individuals revealed that POI samples had a large population of GCs. Moreover, samples from normal individuals exhibited equal proportions of T cells and neutrophils, while samples from AMA women mainly contained MPs (Figure 1D, Table 2).

**Table 1:** The clinical information of the nine individuals

No.	Age	BMI	bFSH	AMH	Oocyte number	Fertilization mode
Normal 1	32	21.3	5.3	2.8	14	IVF
Normal 2	28	22.0	6.8	2.3	16	IVF
Normal 3	30	22.3	6.2	2.7	12	ICSI
AMA 1	41	23.2	13.0	1.2	5	ICSI
AMA 2	43	22.9	19.0	1.1	8	ICSI
AMA 3	45	23.0	20.0	1.5	4	IVF
POI 1	35	23	25.1	0.8	2	ICSI
POI 2	33	22.5	26.0	0.9	1	IVF
POI 3	31	21.8	28.0	0.5	1	IVF

AMA, individuals with advanced maternal age, ≥40 years old; BMI, body mass index; bFSH, basal follicle-stimulating hormone; AMH, anti-Müllerian hormone; IVF, in vitro fertilization; ICSI, intracytoplasmic sperm injection.

**Table 2:** Cell proportion data for each individual

No.	B Cells	Basophils	Granulosa cells	MPs	Neutrophils	T Cells
Normal 1	0.00202	0.000238	0.197386	0.322519	0.424124	0.053714
Normal 2	0.018737	0.001606	0.134368	0.235011	0.220557	0.389722
Normal 3	0.018548	0.001432	0.076196	0.139215	0.306574	0.458035
AMA 1	0.008349	0.00063	0.029773	0.362634	0.390989	0.207624
AMA 2	0.005256	0	0.07972	0.803767	0.061323	0.049934
AMA 3	0.003996	0.006085	0.384378	0.211172	0.187557	0.206812
POI 1	0.010219	0.001298	0.717275	0.055474	0.138848	0.076886
POI 2	0.010004	0.000449	0.172321	0.198422	0.274482	0.344321
POI 3	0.00662	0.000828	0.72476	0.029295	0.115856	0.122642

AMA, individuals with advanced maternal age, ≥40 years old.

### Single-cell RNA sequencing reveals GC heterogeneity across normal individuals, AMA individuals, and POI patients

**Figure 2:**
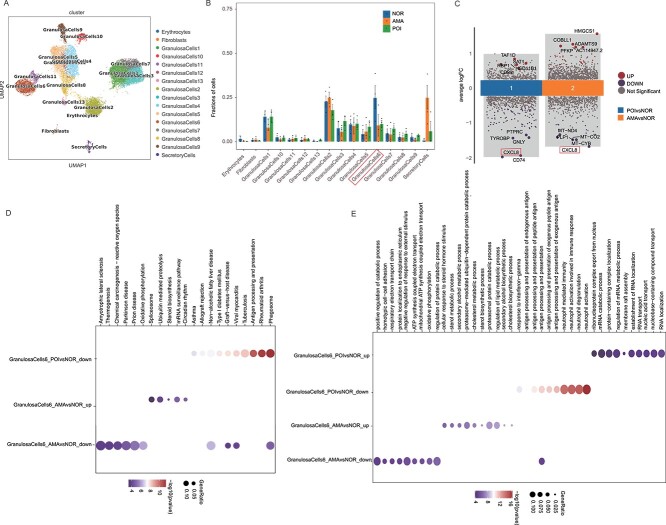
GC subclusters in normal individuals, AMA individuals, and POI patients. (**A**) UMAP plot of the 13 GC subtypes in the nine samples. (**B**) Line graph of the proportions of the 13 GC subclusters in normal individuals, AMA individuals, and POI patients. (**C**) DEGs identified in the GCs in cluster 6 between samples from AMA individuals and POI patients vs. normal individuals. (**D**–**E**) KEGG and GO analyses of the DEGs that were identified in the GCs in cluster 6 in samples from AMA individuals and POI patients vs. normal individuals.

To further investigate their associations with POI pathogenesis, we grouped granulosa cells, T cells, and MPs into subclusters. The granulosa cells were divided into 13 subclusters based on gene expression profiles (Figure 2A). Detailly, cluster 1 was characterized by the high expression of *UGP2*, *PTGES*, *CPM*, *CITED1*, and *STEAP1*; clusters 2 and 3 were highly expressed *MT−RNR1* and *CCKBR*, respectively; cluster 4 was featured with the high expression of *UGP2* and *AREG*; cluster 5 mainly expressed *FAM104A*, *RASD1*, *CCNG1*, *SFRP5*, *CPM*, *CITED1*, and *UGP2*; and cluster 6 was characterized by high expression levels of *MALAT1*, *NEAT1*, *XIST*, *KCNQ1OT1*, and *AC016831.5*, and the features of other clusters are shown in Supplementary Figure S1. Among the 13 groups of granulosa cells, the percentage of cluster 6 was relatively high and showed a same trend in the comparison of the POI versus normal and AMA versus normal group (Figure 2B). Thus, cluster 6 was chosen for further analysis. Analysis of the DEGs of cluster 6 revealed that there were 120 DEGs between the POI group and normal individuals; of these DEGs, 7 (SAT1, *HNRNPA2B1*, *CD99*, *ARGLU1*, *HSD11B1*, *RBP1*, and *TAF1D*) were upregulated and 113 were downregulated. Similar analysis revealed 72 DEGs between the AMA individuals and the normal individuals, 47 of which were upregulated and 25 of which were downregulated. Among the downregulated DEGs, 15 (*CSF3R*, *PI3*, *CXCL8*, *GNLY*, *FCGR3B*, *ITGAX*, *IFITM2*, *GSTA1*, *NEAT1*, *PFN1*, *MT-CO2*, *PLP1*, *HSD3B2*, *MT-CO3*, and *MTATP6P1*) were also present in the DEGs between samples from POI patients and AMA individuals, whereas *SAT1* was the only commonly upregulated gene between these groups (Figure 2C). Moreover, Kyoto Encyclopedia of Genes and Genomes (KEGG) pathway analysis revealed that the phagosome pathway was enriched in the genes whose expression was downregulated in both POI patient and AMA individual samples compared with the normal individuals (Figure 2D), while GO analysis revealed that antigen processing and presentation pathway were enriched in the genes (Figure 2E). These results indicate that POI and natural ovary aging share a common phenotype (e.g. phagosome and antigen processing and presentation).

**Figure 3:**
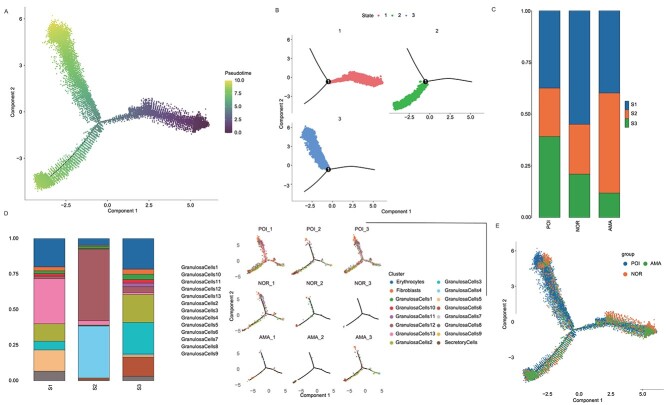
Pseudotime analysis of GCs from normal individuals, AMA individuals, and POI patients. (**A**) Pseudotime analysis reveals the progression of GCs. (**B**) Trajectory reconstruction of all single cells reveals three branches: states 1, 2, and 3. (**C**) The composition of cells in states 1–3. (**D**–**E**) The proportions of cells in the three states in samples from normal individuals, AMA individuals, and POI patients.

Additionally, pseudotime analysis revealed that the pseudotime trajectory began with state 1 and then split into two main branches, state 2 and state 3 (Figure 3A–B). State 2 cells, which were derived from state 1, were the most common in AMA patients. State 1, which was predicted to contain the most primitive cells and was composed mainly of GC subpopulations 1, 3, and 7, was the most common state in normal individuals (Figure 3C–E). In samples from POI patients, state 3 cells, which mainly contained GC subtypes 1, 4, and 5, were the most common (Figure 3C–E). Notably, 35%–40% of GCs in cluster 6 were in state 2, 2% were in state 3, and 0% were in state 1 (Figure 3C). Taken together, these results indicate that the progression from state 1 to state 2/3 caused ovarian dysregulation and that the development of state 2 cells was the main cause of ovarian aging, while the development of state 3 cells was the main cause of POI.

### Single-cell RNA sequencing reveals NK/T-cell heterogeneity across normal individuals, AMA individuals, and POI patients

**Figure 4:**
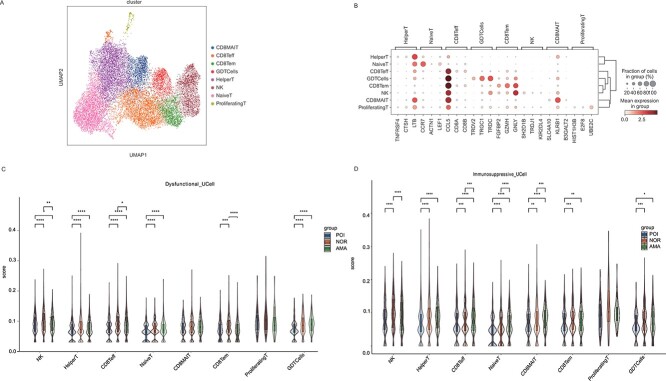
NKT cell subclusters in samples from normal individuals, AMA individuals, and POI patients. (**A**) UMAP plot of the eight NKT cell subtypes in the nine samples. (**B**) Bubble plot of canonical cell type markers of the eight main NKT cell types. (**C**–**D**) The dysfunctional and immunosuppressive scores of the eight NKT cell subclusters in samples from normal individuals, AMA individuals, and POI patients. ^*^P < 0.05, ^**^P < 0.01, ^***^, ^*****^P < 0.001.

We also divided T cells into CD8MAIT (Mucosal Associated Invariant CD8^+^T cells), CD8Teff (Effector CD8^+^ T cells), CD8Tem (Effector memory CD8^+^T cells), GDTCells (gamma delta T cells, γδT cells), HelperT, NativeT, and proliferatingT cell subclusters based on gene expression (Figure 4A). Specifically, CD8MAIT cells expressed *RB1*, CD8Teff cells expressed *CCL5*, CD8Tem cells expressed *GZMH* and *NLY*, GDT cells expressed *TRGC1* and *TRDC*, helper T cells expressed *LTP*, native T cells expressed *CCR7*, and proliferating T cells expressed *UBE2C* (Figure 4B). Analysis of the functional differences between these subclusters in samples from normal individuals, AMA individuals, and POI patients revealed that the dysfunctional signature scores of the NK, CD8Teff, and CD8Tem subgroups, as well as the immunosuppressive signature scores of NK cells, were significantly lower in samples from AMA individuals and POI patients (Figure 4C–D). These results suggest that NK, CD8Teff, and CD8Tem cells were activated in AMA individuals and POI patients.

**Figure 5:**
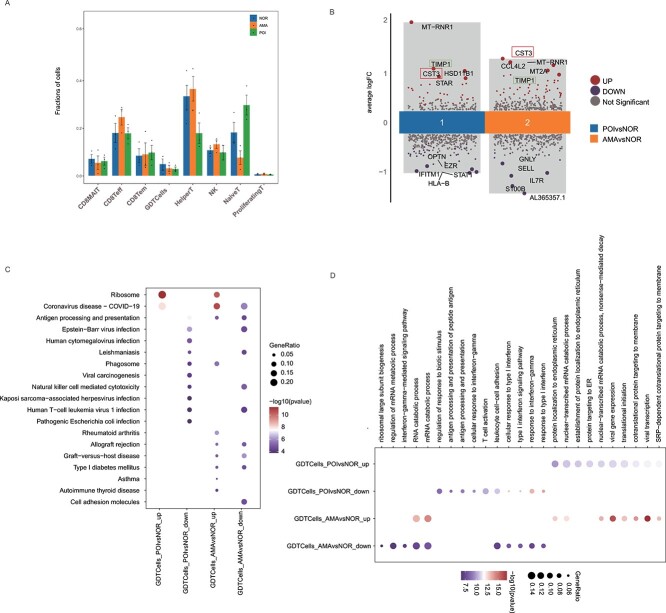
NKT subcluster difference and the pathway enrichment analysis. (**A**) Line graph of the proportions of the eight NKT subclusters in samples from normal individuals, AMA individuals, and POI patients. (**B**) DEGs identified in the GDT cells in samples from AMA individuals and POI patients vs. normal individuals. (**C**–**D**) KEGG and GO analyses of the DEGs identified in GDT cells from AMA individuals and POI patients vs. normal individuals.

Moreover, a comparison of the proportions of these subclusters in samples from normal individuals, AMA individuals, and POI patients revealed that the proportion of GDT cells was lower in both AMA individuals and POI patients (Figure 5A). There were 71 DEGs in between AMA individuals and normal individuals (49 upregulated and 22 downregulated). Moreover, 84 DEGs were identified between POI patients and normal individuals (31 upregulated and 53 downregulated). Among the downregulated DEGs, 14 (*HLA-B*, *EZR*, *MTATP6P1*, *IFITM1*, *RPS26*, *RPL5*, *HLA-E*, *PIP4K2A*, *HNRNPA2B1*, *ETS1*, *AL365357.1*, *HIST1H4C*, *SELL*, and *PRMT2*) were common in the samples from POI patients and AMA individuals. Additionally, 16 upregulated DEGs (*MT-RNR1*, *MT-CO1*, *STAR*, *TIMP1*, *RBP1*, *FTL*, *CST3*, *RPL27A*, *FOS*, *RPS28*, *CCL4*, *C1orf56*, *DHCR24*, *CXCR4*, *BEST1*, and *RGS2*) were found in both of these groups (Figure 5B). KEGG pathway analysis of samples from AMA individuals and POI patients revealed the enrichment of the commonly upregulated genes in ribosomal factors and the enrichment of the commonly downregulated genes in natural killer cell–mediated cytotoxicity and cell adhesion molecules (Figure 5C). Gene Ontology analysis revealed that protein localization at the endoplasmic reticulum (ER) and nuclear-transcribed mRNA catabolic process pathways were enriched in the upregulated genes and that response to interferon-gamma, response to type l interferon, cellular response to type l interferon, and leukocyte cell–cell adhesion pathways were enriched in the downregulated genes (Figure 5D). These results indicate that immune activation and ER-related signaling may be involved in POI pathogenesis and natural ovarian aging.

### Single-cell RNA sequencing reveals MP heterogeneity across normal individuals, AMA individuals, and POI patients

**Figure 6:**
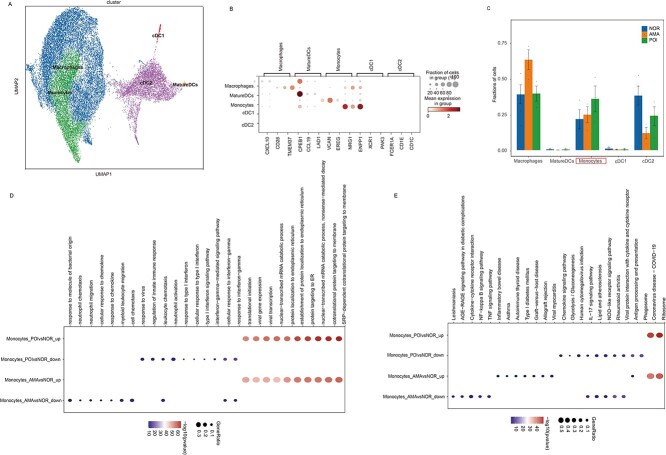
MP subclusters in samples from normal individuals, AMA individuals, and POI patients. (**A**) UMAP plot of the five MP subtypes in the nine samples. (**B**) Bubble plot of canonical cell type markers of the five main MP cell types. (**C**) Line graph of the proportions of the five MP cell subclusters in samples from normal individuals, AMA individuals, and POI patients. (**D**–**E**) GO and KEGG analyses of the differentially expressed genes identified in monocytes from AMA individuals and POI patients vs. the normal group.

To further identify common characteristics between POI patients and AMA individuals, MP cells were divided into macrophage, mature dendritic cell (DC), monocyte, cDC1, and cDC2 subclusters (Figure 6A–B). “Mature” dendritic cells are professional antigen-presenting cells that can activate and regulate immune responses. Dendritic cells are mainly divided into two categories, plasma-like dendritic cell (pDCs) and classical dendritic cells (cDCs), according to the surface markers and functional features. Also, cDCs are further subdivided into type I classic dendritic cells (cDC1) and type II classic dendritic cells (cDC2), both differentiating from a common DC precursor, and eventually developing to mature DCs to activate T cells [23]. The current data revealed that the monocyte population was greater in samples from POI patients and AMA individuals than in those from normal individuals (Figure 6C). A total of 265 DEGs (103 upregulated and 162 downregulated) were identified in samples from AMA individuals, whereas 166 upregulated genes and 234 downregulated genes were identified in samples from POI patients. Pathway enrichment analysis of the DEGs that were identified in samples from POI patients and AMA individuals compared with normal individuals revealed that nonsense-mediated decay protein targeting to the ER, establishment of protein localization to the ER, protein localization to the ER, and nuclear-transcribed mRNA catabolic process were enriched in the GO analysis, revealing that leukocyte chemotaxis was enriched in the downregulated genes (Figure 6D). KEGG pathway analysis revealed that in samples from AMA individuals and POI patients, ribosomal pathways were enriched in the upregulated genes, and antigen processing and presentation pathways were enriched in the downregulated genes (Figure 6E). We hypothesize that a higher number of ribosomes may expand the ER membrane and induce inflammation, thereby driving POI and natural ovarian aging.

### Loss of the VEGFA–FLT1 interaction between monocytes and GCs may contribute to POI pathogenesis

**Figure 7:**
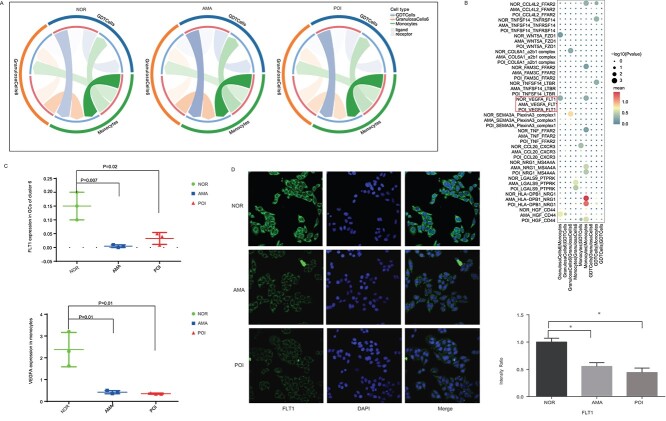
Cell–cell interactions in samples from normal individuals, AMA individuals, and POI patients. (**A**) The ligand–receptor interaction landscapes between GDT cells, monocytes, and GCs in cluster 6 in samples from the three groups. (**B**) Ligand–receptor interactions revealed marked differences between samples from normal individuals and POI patients. (**C**) The expression levels of FLT1 in GCs of cluster 6 and VEGFA in monocytes of the 9 individuals based on the scRNA-seq are shown. (**D**) The expression of FLT1 in human GCs from the three groups was detected using immunofluorescence staining.

Next, we examined the cell–cell interactions between GDT cells, monocytes, and GCs in cluster 6. The number of cell–cell interactions between the three cell types did not appear to vary across samples from normal individuals, AMA individuals, and POI patients (Figure 7A). We further analyzed the detailed differences and density between the three cell types in samples from normal individuals, AMA individuals, and POI patients. This analysis showed that some ligand–receptor pairs, including VEGFA–FLT1, CCL4L2–FFAR2, TNFSF14–TNFRSF14, and WNT5A–FZD1, were present only in normal individuals, whereas others, such as NRG1–MS4A4A, LGALS9–PTPRK, and HLA−DPB1–NRG1, were present only in samples from AMA individuals and POI patients (Figure 7B). The VEGFA–FLT1 signaling axis stimulates seemingly noninflammatory and inflammatory responses in various tissues and involves a variety of biological processes, including angiogenesis, which has been verified to be closely involved in the regulation of ovarian function [24–26]. For example, Zhou et al. demonstrated that Gui ShenWan, a Chinese medicine, prevented premature ovarian insufficiency through promoting the expression of VEGFR [24]. Thus, we focused on investigating the interaction of VEGFA-FLT1, and assessed the expression levels of FLT1 and VEGFA in GCs derived from POI patients, AMA, and normal individuals based on the scRNA-seq data. The results showed that the expression levels of FLT1 and VEGRA were significantly lower in POI patients and AMA individuals than that of the normal individuals (Figure 7C), as well as the FLT1 expression detected by the immunofluorescence (Figure 7D). These results suggest that the loss of the VEGFA–FLT1 interaction between monocytes and GCs may contribute to POI pathogenesis.

## Discussion

In this study, we revealed that loss of the VEGFA–FLT1 interaction in monocytes and granulosa cells, along with enriched ER- and ribosome-related pathways, may drive excess inflammation, accelerating granulosa cell senility and contributing to infertility.

Under physiological conditions, adequate inflammatory stress is necessary for normal follicular development and ovulation [27]. Before ovulation, GCs possess an inflammatory and immune-like phenotype that produces prostaglandins, inflammatory cytokines, and chemokines, which promote ovulation and fertilization [28]. However, ovarian biopsies of POI patients have shown elevated lymphocyte infiltration and immune responses, and excess inflammation might contribute to ovarian aging and the development of POI [29]. The proportion of GCs in cluster 6, which are characterized by high expression levels of *MALAT1*, *NEAT1*, *XIST,* and *KCNQ1OT1*, was greater in samples from AMA individuals and POI patients. Most of these genes, including *MALAT1* [30], *KCNQ1OT1* [31], and *NEAT1* [32], are implicated in inflammatory responses. The expression of *KCNQ1OT1*, which is also involved in DNA methylation, is also reported to be significantly reduced in vitrified oocytes [33], suggesting that *KCNQ1OT1* may play a role in the regulation of ovarian function. We found that phagosome and antigen processing and presentation pathways were enriched in the DEGs identified in GCs in cluster 6 between the AMA individuals and POI patients and normal individuals. Similarly, Wang et al. [34] compared protein expression in ovarian follicular fluid and cumulus cells from patients with a diminished ovarian response and healthy controls and found that the differentially expressed proteins were mostly enriched in the phagosome process. Although antigen processing and presentation are immune-related pathways, they are also implicated in the regulation of ovarian follicle development [35]. Our findings suggest that excess inflammation plays a crucial role in both ovarian aging and POI. This possibility was further supported by observations that the dysfunctional signature scores of T-cell subtypes, such as the NK, CD8Teff, and CD8Tem subgroups, and the immunosuppressive signature scores of NK cells were significantly lower in samples from AMA individuals and POI patients.

Additionally, we found that the proportion of GDT cells, which express high levels of *TRGC1* and *TRDC*, was decreased in samples from AMA individuals and POI patients. GDT means gamma delta T cells, which, together with alpha-beta (αβ) T cells, represent two different T-cell lineages that have been defined by their expression of αβ or γδ T-cell receptors. Although GDT cells share many effector capabilities with αβ T cells (e.g. cytotoxicity and cytokine production), the lineages exhibit different biological properties, such as thymic-dependent or -independent development, major histocompatibility complex restriction, and recognition of soluble protein and non-protein antigens of endogenous origin, endowing some advantages for it particularly of cell therapy [36–38]. Here, ribosomal factors and protein localization to the ER were enriched in the genes that were found to be upregulated in GDT cells from AMA individuals and POI patients compared with normal individuals. Consistently, ribosomal factors and ER-related pathways, such as nonsense-mediated decay protein targeting to the ER, establishment of protein localization to the ER, and protein localization to the ER, were enriched in the genes whose expression was upregulated in monocytes from AMA individuals and POI patients compared with monocytes from normal individuals. The ribosome is a multiunit complex that translates mRNA into protein. Ribosomal biogenesis plays an essential role in cell proliferation, differentiation, apoptosis, development, and transformation [39]. Mounting evidence indicates that interference with ribosomal biogenesis causes cancer, aging, and age-related degenerative diseases [39, 40]. A recent study revealed that the ribosomes of aging cells move more slowly and periodically, thereby “stalling” translation and causing ribosome collisions and the accumulation of new peptides, which contributes to ribosomal dysfunction and accelerates aging [41]. Based on single-cell quantification of ribosome occupancy and proteomics data, Ozadam et al. [42] recently reported that ribosome occupancy in germinal vesicle-stage oocytes is the primary determinant of protein abundance in the zygote. The ER plays a crucial role in triggering the deterioration of oocyte quality during oocyte aging [43–45]. Interestingly, ribosome-rich ER membranes expand, which might promote TNF biosynthesis [46]. Thus, we hypothesize that high ribosome numbers may cause ER expansion, thereby triggering inflammation and contributing to natural ovarian aging and POI development.

Moreover, we observed that the VEGFA–FLT1 interaction between monocytes and GCs was absent in samples from AMA individuals and POI patients. Fms-like tyrosine kinase-1 (FLT1), also known as VEGFR1, is abundantly expressed on the membranes of monocytes and macrophages and transduces signals for migration and cytokine/chemokine production by these cells. The VEGF–FLT1 axis stimulates seemingly noninflammatory and inflammatory responses in various tissues and promotes the development of a variety of diseases, including cancer (via angiogenesis and lymphangiogenesis), arthritis, and atherosclerosis [47]. Also, accumulated evidence has demonstrated that vascular regeneration is closely involved in the pathogenesis of POI [24–26]. Detailly, Qu et al. [25] reported that miR-126-3p containing exosomes derived from human umbilical cord mesenchymal stem cells promote angiogenesis through promoting the expression of VEGF and attenuate ovarian granulosa cell apoptosis in a preclinical rat model of POI. In addition, Ovayolu et al. [48] found that the level of soluble fms-like tyrosine kinase receptor-1 (sFlt-1) was decreased in the blood samples of POI women as compared with that of the healthy women. Herein, we think that the monocytes and granular cells adhere to each other via FLT1–VEGFA axis may also contribute to the angiogenesis and thereafter maintain the ovarian function under normal conditions. Taken together, we hypothesize that the loss of the VEGFA–FLT1 interaction, along with the enriched ER and ribosomal pathways, may drive excess inflammation, which accelerates GC senility and the state of infertility. A limitation of this study is that we did not use in vivo and/or in vitro experiments to validate this hypothesis.

In this study, we analyzed the cellular landscape of follicular using follicular fluid samples from normal individuals, AMA individuals, and POI patients, and cells such as T cells, B cells, neutrophils, basophils, MPs, and GCs were present. Further analysis revealed a common follicle immune cell constitution in POI patients and AMA individuals, including a greater proportion of GCs in cluster 6 and monocytes and a lower proportion of GDT cells than those in the follicular environment of normal individuals. We hypothesize that the loss of the VEGFA–FLT1 interaction in monocytes and GCs, along with enriched ER and ribosomal pathways, may contribute to excess inflammation, which accelerates GC senility and contributes to infertility. This study provides novel insights into the pathogenesis of POI and aging and highlights targeting the VEGFA–FLT1 interaction may be a potential strategy for inhibiting inflammation and treating POI, as well as delaying senescence.

## Supplementary Material

Supplementary_Figure_1_ioae157

Supplementary_figure_ioae157

## Data Availability

Publicly available datasets were analyzed in this study. The raw data have been uploaded to the Genome Sequence Archive for Human (https://ngdc.cncb.ac.cn/gsa-human/submit/hra/subHRA007647/finishedOverview). Other data will be made available to the editors of the journal for review or upon request.
