# Supplementary figures and images for: Single-cell RNA sequencing reveals common interactions between follicle immune cells and granulosa cells in premature ovarian insufficiency patients
Source: Biol Reprod. 2024 Nov 8;112(1):156–68. doi: 10.1093/biolre/ioae157 (PMC11736418; doi:10.1093/biolre/ioae157)

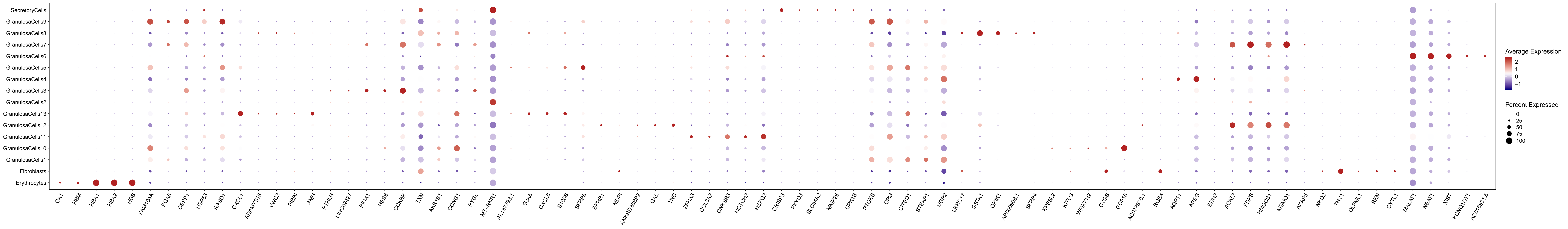

Supplement: Supplementary_Figure_1_ioae157 [file supplementary_figure_1_ioae157.pdf]
