# Supplementary material for: Single-cell RNA sequencing reveals common interactions between follicle immune cells and granulosa cells in premature ovarian insufficiency patients
Source: Biol Reprod. 2024 Nov 8;112(1):156–68. doi: 10.1093/biolre/ioae157 (PMC11736418; doi:10.1093/biolre/ioae157)
Supplement: Supplementary_figure_ioae157 [file supplementary_figure_ioae157.docx]

**Supplementary figure 1.** Dot plot of the top markers of the 13 GC subtypes in the nine samples.
